# The Duration of Syphilogenic Capacity in Relation to Marriage

**Published:** 1887-07

**Authors:** 


### Selections.

---

### *THE DURATION OF THE SYPHILOGENIC CAPACITY IN RELATION TO MARRIAGE.*

Syphilis differs from other infectious diseases in its prolonged virulence and susceptibility of hereditary transmission. The influence of both of these characteristics is modified during the course of the disease. As the disease advances, the virulent principle gradually loses its force and finally becomes exhausted. The length of time during which the disease may be communicated by direct contagion or by hereditary transmission makes the question of marriage a most important one, both from a medical and social standpoint. The question is often asked the physician, when may a syphilitic man marry with safety? The answer to this question not only involves the happiness and welfare of man and wife, but the possible offspring of this marriage union.

At one time the majority of syphilographers asserted that the contagious activity of syphilis began and ended with the chancre, but this view has been abandoned and it is now generally held that contagion ends with secondary lesions.

Dr. Otis divides the disease into two distinct stages, the one active, including the so-called primary and secondary stage and the second passive or so-called tertiary stage lacking in the contagious element; whilst Dr. Morrow asserts that secondary accidents may continue to recur for months or years after the chronological completion of the secondary stage with all the contagious property of that stage.

In other words, Dr. Morrow holds that the disappearance of the virulent principle does not always take place with the chronological completion of the secondary stage, but is extended into the tertiary stage. Dr. Morrow's views are based upon clinical experience, which shows that late lesions are exceptionally, but none the less certainly, the source of contagion. Holding this opinion, Dr. Morrow asserts that the contagious activity of syphilitic virus and its susceptibility of hereditary transmission cannot be considered to have ceased after a given period of time, as, for example, after the third or fourth year, whilst, as opposed to this opinion, Dr. Otis fixes the arbitrary period of three years, or at most at four years, as definitely marking the end of the contagious stage of syphilis in all cases. According to Dr. Otis' view, in the vast majority of cases there is a practical termination of the contagious stage of syphilis in three years, and if the patient has been persistently treated during this time, he regards marriage as permissible. Dr. R. W. Taylor believes that no law can ever be made defining with mathematical certainty just when syphilis will cease to be contagious. He thinks the keynote of the contagiousness of syphilis can be treated in one word, treatment. "If a case of syphilis is taken early, and treated from the beginning of the secondary manifestations for two years, in the great majority its contagious character will disappear within that period or within two years and a-half." It is thus shown that eminent syphilographers are not fully in accord upon this point. It is quite certain that the duration of the syphilogenic capacity in relation to the marriage cannot be wholly determined by arbitrary periods of time, inasmuch as the contagious principle and hereditary influence are both influenced by the manner of treatment and condition of the syphilitic subject at the time of marriage.

The discussion of this subject, published in the *Journal of Cutaneous and Genito-Urinary Disease*, (April, 1887,) is worthy of study by those interested in this branch of medical work. We present the following brief survey of the subject formulated by Dr. Morrow:

"1. The facts of every-day observations show that there is nothing constant in contagion, nothing certain in heredity. Many men marry with a syphilis in full activity of secondary manifestation and never infect their wives or transmit the disease to their offspring. These negative observations are, however, entirely valueless as a basis for estimating positive result

"2. The modern division of syphilis into secondary and tertiary periods, based upon anatomical forms and processes, does not furnish a safe criterion for determining the contagious character of the lesions.

"3. The chronological completion of the secondary stage does not always mark the definite disappearance of the virulent principle; clinical experience shows that late lesions are exceptionally, but none the less certainly, the source of contagion.

"4. While in the immense majority of cases the contagious activity of syphilis and its susceptibility of hereditary transmission cease after the third or fourth year, yet well-authenticated observations prove in the most positive manner that these qualities sometimes continue in force much longer and may be manifest in the fifth and sixth years of the disease, and even later.

"5. The aptitude of syphilitic parents to procreate diseased children may persist after the cessation of all specific manifestations; the contagious stage of syphilis is not, therefore, the exact measure of the duration of hereditary influence.

"6. The precise date in the evolution of the diathesis when the syphilitic organism undergoes that radical transformation which marks the limit of its contagious or transmissive power, does not admit of mathematical expression.

"7. It is probable that this limit varies in different cases and that many circumstances contribute to advance or defer it.

"8. The type of the syphilis, the constitutional peculiarities of the patient, the character of the treatment, the presence or absence of certain conditions which are recognized as factors of gravity in syphilis, all exert a modifying influence.

"9. All these elements should be taken into consideration in deciding upon the admissibility of a syphilitic man to marriage; each case should be studied upon its individual merits.

"10. The direct paternal transmission of syphilis without preliminary infection of the mother, may be classed among the most conclusively established facts of medical science.

"11. It is, therefore, a dangerous doctrine to teach that the sole risks a syphilitic man introduces into marriage consist in the contagious accident he may bear upon his person.

"12. The arbitrary designation of a limit of three, or at most four years, as perfectly safe for a syphilitic man to marry, with or without treatment and irrespective of the actual existence of specific lesions, is unwarranted by science or the teachings of experience.

"The conditions of admissibility to marriage formulated by Fournier are much broader, more scientific, more safe. These demand a mild or medium type of the disease, an advanced age of the diathesis, three or four years at the minimum, and a prolonged immunity, eighteen months to two years, from specific accidents; if these guarantees of safety are further fortified by sufficient specific treatment, a reluctant consent is given; marriage is tolerated rather than advised."—*Maryland Medical Journal.*

